# A Review of the Efficacy of FDA-Approved *B. anthracis* Anti-Toxin Agents When Combined with Antibiotic or Hemodynamic Support in Infection- or Toxin-Challenged Preclinical Models

**DOI:** 10.3390/toxins13010053

**Published:** 2021-01-13

**Authors:** Zoe Couse, Xizhong Cui, Yan Li, Mahtab Moayeri, Stephen Leppla, Peter Q. Eichacker

**Affiliations:** 1 Critical Care Medicine Department, Clinical Center, National Institutes of Health, Bethesda, MD 20892, USA; zoe.mendrysa@cc.nih.gov (Z.C.); cxizhong@cc.nih.gov (X.C.); yli3@cc.nih.gov (Y.L.); 2 National Institutes of Allergy and Infectious Diseases, National Institutes of Health, Bethesda, MD 20892, USA; mmoayeri@niaid.nih.gov (M.M.); sleppla@niaid.nih.gov (S.L.)

**Keywords:** *B. anthracis*, anthrax, lethal and edema toxin, shock, antibiotic support, hemodynamic support, anti-toxin

## Abstract

Anti-toxin agents for severe *B. anthracis* infection will only be effective if they add to the benefit of the two mainstays of septic shock management, antibiotic therapy and titrated hemodynamic support. Both of these standard therapies could negate benefits related to anti-toxin treatment. At present, three anthrax anti-toxin antibody preparations have received US Food and Drug Administration (FDA) approval: Raxibacumab, Anthrax Immune Globulin Intravenous (AIGIV) and ETI-204. Each agent is directed at the protective antigen component of lethal and edema toxin. All three agents were compared to placebo in antibiotic-treated animal models of live *B. anthracis* infection, and Raxibacumab and AIGIV were compared to placebo when combined with standard hemodynamic support in a 96 h canine model of anthrax toxin-associated shock. However, only AIG has actually been administered to a group of infected patients, and this experience was not controlled and offers little insight into the efficacy of the agents. To provide a broader view of the potential effectiveness of these agents, this review examines the controlled preclinical experience either in antibiotic-treated *B. anthracis* models or in titrated hemodynamic-supported toxin-challenged canines. The strength and weaknesses of these preclinical experiences are discussed.

## 1. Introduction

While *B. anthracis* infection (anthrax) is predominately a health problem in underdeveloped regions, the developed world must contend with it as well. The 2001 US outbreak with both inhalational and cutaneous forms of infection highlighted the potential weaponization of anthrax spores [1]. A subsequent outbreak of soft-tissue infection due to contaminated heroin use in injection drug users in the United Kingdom and Europe between 2009 and 2011, with more than 50 confirmed and more probable cases, again emphasized the bacterium’s threat [2,3]. Notably, these outbreaks suggested that shock with invasive forms of anthrax was more difficult to treat than with more commonly encountered bacteria. *B. anthracis* continues to be a Category A and Tier 1 select agent and potential bioweapon, presenting the highest risk to the US public and requiring the highest level of concern and preparedness [4]. 

The pathogenesis of anthrax is closely associated with the bacterium’s lethal (LT) and edema (ET) toxins [5,6,7]. These are binary toxins consisting of protective antigen (PA), the component necessary for host cell uptake of the toxins’ toxic moieties: lethal factor (LF) for LT and edema factor (EF) for ET. Lethal factor is a protease that cleaves mitogen-activated protein kinase kinases and stimulates inflammasome activity [8]. Edema factor has adenylate-cyclase activity and increases intracellular 3′,5′-cyclic adenosine monophosphate (cAMP) levels [9,10]. Following the 2001 US anthrax attack and based on the central pathogenic roles LT and ET have in *B. anthracis* infection, efforts to develop therapies neutralizing the toxins or their actions accelerated and focused largely on development of antibodies to the toxins. 

Anti-toxin agents for anthrax-associated shock will only be effective if they add to the benefit of the two mainstays of sepsis and septic shock management: antibiotic therapy and titrated hemodynamic support [11,12]. Both of these standard therapies could negate benefits related to anti-toxin treatment. At present, three anthrax anti-toxin antibody preparations have received US Food and Drug Administration (FDA) approval: ABthrax (Raxibacumab, Emergent BioSolutions, Rockville, MD, USA), Anthrax Immune Globulin Intravenous (AIGIV, or Anthrasil, Emergent BioSolutions, Rockville, MD, USA) and ETI-204 (Elusys Therapeutics, Parsippany Troy-Hills, NJ, USA) [13,14,15]. Each agent is directed at the PA component of LT and ET. All three agents were reported to be effective when compared to placebo controls in antibiotic-treated animal models of live *B. anthracis* infection [15]. In each case, experiments were done which tested the effects of the agents when administered following bacterial challenge. But none of these studies examined whether the antitoxins improved outcomes when combined with standard fluid and vasopressor hemodynamic support (HS). This supportive measure must be continuously titrated and could not be reliably administered in a live *B. anthracis* study conducted under Biosafety Level-3 conditions. However, Raxibacumab and AIG have also been shown to be effective when combined with standard hemodynamic support in a 96 h canine model of anthrax toxin-associated shock conducted at Biosafety Level-2 [16,17,18].

Of the three anthrax anti-toxin agents now approved for use in the US, only AIG has actually been administered to a group of infected patients. This experience was not a controlled one, consisting of the agent’s administration in a subset of patients during the UK anthrax outbreak in injection drug users, and it offers little insight into the efficacy of the agents [1]. It is also likely that for proprietary reasons and because severe anthrax infection is infrequent, these three FDA-approved anti-toxin agents will never be directly compared. Therefore, to provide a comparison of the potential efficacy of these FDA-approved agents, the present review summarizes the controlled preclinical experience with them when combined either with antibiotic treatment in *B. anthracis*-challenged models or with titrated hemodynamic support in the toxin-challenged canine model. Only studies examining anti-toxin treatment at the time of or following bacteria or toxin challenge are reviewed. The strengths and weaknesses of these preclinical experiences are then discussed.

## 2. Effects of the Three *B. anthracis* Anti-Toxin Agents when Combined with Antibiotics in Live *B. anthracis* Challenged Animal Models

Raxibacumab was the first anthrax anti-toxin agent approved by the FDA, followed by AIGIV and then ETI-204. As part of a larger previously published systematic review and meta-analysis, we examined the antibiotic-treated *B. anthracis* challenged animal studies provided to support the approval of the three agents [15]. Each agent was tested in preclinical models with inhalational *B. anthracis* Ames strain challenges, but the doses of bacteria, the antibiotics employed, and the timing of therapy differed (Table 1 and Table 2, adapted from Reference [15]). For each agent, some experiments employed rabbits and some cynomolgus macaques (cynos). Data was available for each agent either from published reports or from FDA briefing documents. The effect of treatment on survival was the primary endpoint in these studies. All reports provided numbers of animals living or dying in experiments, but survival curves were only available for three. Relative risks (RR) of death (95% CI) were previously calculated in order to compare treatment effects across studies [15]. Each agent is presented separately here followed by a brief summary of the previously published analysis of the combined experience [15].

### 2.1. Raxibacumab

**Figure 1:**
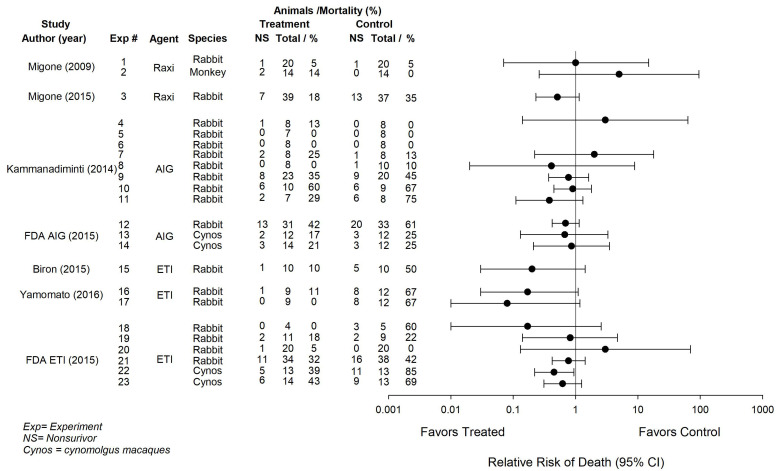
This figure shows the anti-toxin agent and species studied, the numbers of total and non-surviving animals in treatment and control groups with respective mortality rates and the effects of the anti-toxin agent (i.e., agents) on the relative risk of death (95% confidennterval (CI)) for 23 individual experiments from 7 studies. The anti-toxin agents studied included: Raxibacumab (Raxi), Anthrax Immune Globulin (AIG) and ETI-204 (ETI). Control treatments are shown in Table 2. This figure was adapted from Reference [15].

**Figure 2:**
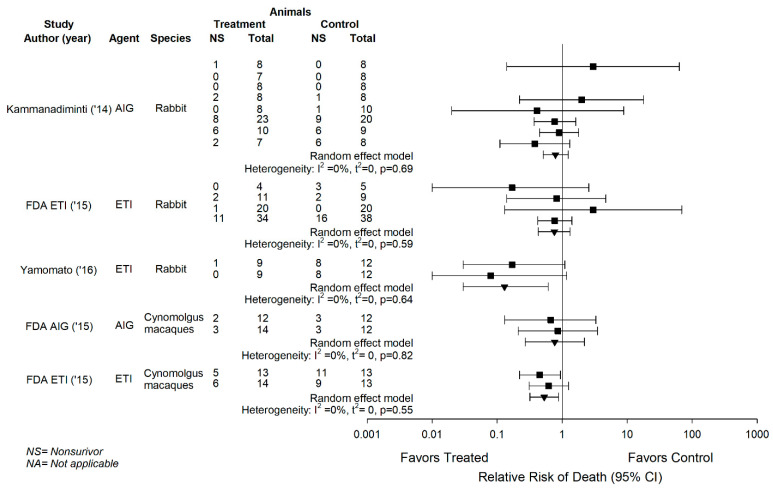
This figure shows data from five studies testing an anti-toxin agent in more than one experiment in the same species, as well as the overall effects anti-toxin agents had on the relative risk (RR) of death (95% CI) across each of these 5 groups of experiments and the I^2^ and its level of significance for the consistency of these overall effects. Individual RRs for experiments are shown by the squares and overall RRs are shown by the inverted triangles. In the five studies shown, Anthrax Immune Globulin (AIG) was studied in eight experiments in the rabbit and two experiments in the cynomolgus macaques, and ETI-204 (ETI) was studied in two experiments in the rabbit and two in the cynomolgus macaques. In the five studies testing AIG or ETI, these agents had very consistent effects on the side of benefit across experiments (I^2^ = 0, *p* ≥ 0.56) in the same species. This figure was adapted from Reference [15].

**Figure 3:**
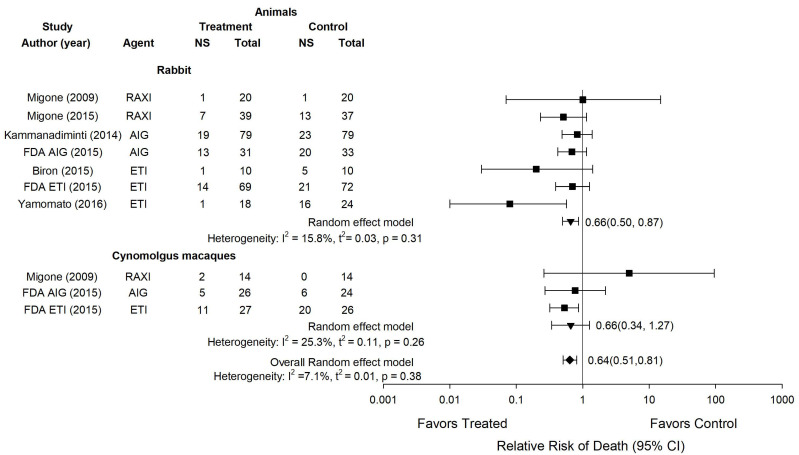
Based on the similarity of the effects of individual anti-toxin agents across experiments in the same species (shown in Figure 2), this figure shows the overall effects of the anti-toxin agents on the relative risk (RR) of death (95% CI) across studies within the same species and the I^2^ and its level of significance for the consistency of these overall effects. Individual RRs for studies are shown by the squares and overall RRs are shown by the inverted triangles. Raxibacumab, AIG and ETI-204 in seven studies in rabbits, and Raxibacumab, AIG and ETI-204 in three studies in cynomolgus macaques, were all associated with reductions in RR. Because the effects of the anti-toxin agents were consistent across these studies in the same species (I^2^ ≤ 25.3%), the effects of treatment on the RR averaged across all anti-toxin agents and species is shown by the diamond at the bottom of the figure. This figure was adapted from Reference [15].

Raxibacumab is a recombinant, fully human, IgG1λ monoclonal antibody against *B. anthracis* PA [19]. Initial data presented to the FDA in support of its approval included two experiments (Experiments 1 and 2 in Table 1 and Table 2 and Figure 1, Figure 2 and Figure 3), one in rabbits (*n* = 40 animals) and one in cynomolgus macaques (*n* = 28) treated with levofloxacin [50 mg/kg, orally per day (PO, qd) for 3 days] or ciprofloxacin (75 mg, PO, qd for 3 days), respectively (Table 1 and Table 2) [19,20,21]. Antibiotics were started in all animals on the day of challenge with 200 × 50% lethal dose (LD50) dose of anthrax spores. On the same day, animals were randomized and received treatment with one dose of either Raxibacumab [40 mg/kg, intravenously (IV); *n* = 20 rabbits and *n* = 14 cynomolgus macaques] or placebo (*n* = 20 rabbits and *n* = 14 cynomolgus macaques) when PA was detected in the blood. At 28 days, Raxibacumab did not alter the RR significantly in either rabbits (1.00 (0.07, 14.90)) or cynomolgus macaques (5.00 (0.26, 95.32)) (Figure 1, adapted from Reference [15]). To more closely simulate the clinical experience, a subsequent study (Experiment 3 in Table 1 and Table 2 and Figure 1, Figure 2 and Figure 3) conducted in rabbits then compared treatment with levofloxacin (50 mg/kg, PO, qd for 3 days) combined with either Raxibacumab (40 mg/kg) or placebo started 84 h after infection with 2.1 × 10^7^ anthrax spores [20]. In order to have sufficient animals still alive to randomize at 84 h, 180 animals were initially challenged. At 84 h, of 76 surviving animals started on levofloxacin, 39 were randomized to receive Raxibacumab and 37 placebo. At 28 days following the final antibiotic treatment, 32 (82%) Raxibacumab and 24 (64.9%) controls had survived (*p* = 0.087), with a RR with Raxibacumab of 0.51 (0.23, 1.14) (Figure 1). This latter study in antibiotic-treated rabbits is referenced in the package insert for the clinical use of Raxibacumab [21]. Data regarding the current Good Manufacturing Practices (cGMP) status of the materials tested was not evident after review of relevant references [21].

### 2.2. Anthrax Immune Globulin Intravenous

Anthrax Immune Globulin Intravenous (AIGIV) is a purified human immuoglobin G (IgG) produced with plasma obtained from healthy donors vaccinated with Anthrax Vaccine Adsorbed (AVA) [15,22,23]. A published study described eight experiments in which rabbits (16 to 72 animals per experiment) were treated with levofloxacin (50 mg/kg, PO, qd for 3 days) combined with either AIGIV (15 U/kg, IV) or placebo, started 30, 36, 48, 60 (2 experiments), 72, 84 or 96 h after inhalational challenge with 2.1 × 10^7^ anthrax spores (Experiments 4 to 11 in Table 1 and Table 2 and Figure 1, Figure 2 and Figure 3) [22]. Survival was monitored for 32 days. All animals died in the AIGIV and placebo groups with treatment started at 36 and 48 h and no RRs were calculated. Otherwise, the RRs with AIGIV were: 3.00 (0.14, 63.74) with 30 h treatment, 2.00 (0.22, 17.89) with 60 h treatment in one experiment and 0.41 (0.02, 8.87) in the other, 0.77 (0.37, 1.62) with 72 h treatment, 0.90 (0.45, 1.79) with 84 h treatment and 0.38 (0.11, 1.31) with 96 h treatment (Figure 1). None of these effects were individually significant. Two additional studies with AIGIV were reported in an FDA briefing document [23]. In the largest experiment with the agent, 96 h after 336 rabbits received an inhalational challenge with 200 × LD50 doses of anthrax spores, the 64 surviving animals were treated with levofloxacin (50 mg/kg, PO, qd for 3 days) combined with either AIG-IV (15 U/kg, IV, *n* = 31) or placebo (*n* = 33) (Experiment 12 in Table 1 and Table 2 and Figure 1, Figure 2 and Figure 3). Survival at 28 days included 18 (58%) AIG-IV versus 13 (39%) placebo animals. The RR [0.69 (0.42, 1.14)] was decreased with AIGIV, but not significantly (Figure 1). The remaining study was conducted in 60 cynomolgus macaques. Sixty-four hours after animals were challenged with 200 × LD50 anthrax spores, surviving animals were treated with ciprofloxacin (32 mg/kg, PO followed by doses of 16 mg/kg, PO, qd) combined with either AIGIV 15 U/kg, IV (*n* = 12), AIGIV 30 U/kg (*n* = 14) or placebo (*n* = 12). Survival at 28 days included 10 animals (83%) receiving AIGIV 15 U/kg, 11 (79%) receiving AIG-IV 30 U/kg and 9 (75%) receiving placebo. Compared to placebo, both AIGIV doses reduced the RR (0.67 (0.13, 3.30) and 0.86 (0.21, 3.48), respectively) but not significantly (Experiments 13 and 14 in Table 1 and Table 2 and Figure 1, Figure 2 and Figure 3). The package insert for AIGIV provides an extensive review of these experiments [23]. Information available indicated that the AIGIV tested was from a facility that had passed an FDA Biologics Team cGMP inspection [23].

### 2.3. ETI-204

ETI-204 is a monoclonal antibody directed against PA. It consists of human constant region sequences and deimmunized murine variable region sequences produced from 1 H, an affinity enhanced recombinant single-chain variable fragment (scFv) derived from the murine mAb 14 B7 [24]. In an initial reported experiment (Experiment 15 in Table 1 and Table 2 and Figure 1, Figure 2 and Figure 3), 20 rabbits received an inhalational challenge with 150–250 × LD50 anthrax spores [24]. When PA was detected in animals or no later than 30 h, animals were treated with doxycycline (2 mg/kg, IV, 2 times per dayfor 3 days) combined with either ETI-204 (8 mg/kg, IV, *n* = 10) or placebo (*n* = 10). Survival at 28 days included 9 animals (90%) receiving ETI-204 and 5 (50%) receiving placebo. ETI-204 reduced the RR, but not significantly [0.20 (0.03, 1.42)] (Figure 1). In a second study, 30 rabbits were challenged with 200 × LD50 anthrax spores [25]. Nine hours following challenge, animals were treated with levofloxacin (50 mg/kg, PO, qd for 5 days) combined with either ETI-204, 4 mg/kg, IV (*n* = 9), ETI-204, 8 mg/kg, IV (*n* = 9) or placebo (*n* = 12). Survival at 34 days included 8 animals (89%) receiving ETI-204, 4 mg/kg, 9 (100%) receiving ET-204, 8 mg/kg, and 4 (33%) receiving placebo. Compared to placebo, both ETI-204 doses reduced the RR (0.17 (0.03, 1.10) and 0.08 (0.01, 1.18), respectively), but not significantly (designated as Experiments 16 and 17 in Table 1 and Table 2 and Figure 1, Figure 2 and Figure 3). Six additional experiments testing ETI-204, four in rabbits and two in cynomolgus macaques, were described in an FDA briefing document [26]. Animals received an inhalational challenge of 200 × LD50 dose of anthrax spores. In three experiments that started with 32, 32 and 40 rabbits, animals were treated with levofloxacin (50 mg/kg, qd for 5 days) started either 96, 72 or 30 h after spore challenge (Experiments 18, 19, and 20 respectively, in Table 1 and Table 2 and Figure 1, Figure 2 and Figure 3). Levofloxacin was combined with either ETI-204 (8 mg/kg, IV, *n* = 4) or placebo (*n* = 5) with 96 h treatment, ETI-204 (8 mg/kg, IV, *n* = 11) or placebo (*n* = 9) with 72 h treatment, or ETI-204 (16 mg/kg, IV, *n* = 20) or placebo (*n* = 20) with 30 h treatment. Survival at 28 days included 4 animals (100%) receiving ETI-204 and 2 (40%) placebo at 96 h (Experiment 18), 9 animals (82%) receiving ETI-204 and 7 (78%) placebo at 72 h (Experiment 19) and 19 animals (95%) receiving ETI-204 and 20 (100%) placebo at 30 h (Experiment 20). The RRs with ETI-204 were variable and none were significant in these three experiments (0.17 (0.01, 2.57), 0.82 (0.14, 4.71) and 3.00 (0.13, 69.42), respectively) (Figure 1). The fourth rabbit experiment included 103 animals (Experiment 21). Seventy-two hours after spore challenge, surviving animals were treated with levofloxacin (6.5 mg/kg, PO, for 3 days) combined with ETI-204 (16 mg/kg, IV, *n* = 34) or placebo (*n* = 38). Survival at 28 days included 23 animals (68%) receiving ETI-204 and 22 (58%) placebo. ETI-204 reduced the RR but not significantly [0.77 (0.42, 1.42)] (Figure 1). The two experiments in cynomolgus macaques were similar to each other. Both started with 32 animals. Forty-eight hours after spore challenge, surviving animals were treated with ciprofloxacin (10 mg/kg, PO for 4 days). Antibiotics were combined with ETI-204 (8 mg/kg, IV, *n* = 13) or placebo (*n* = 13) in one experiment (Experiment 22) and with the same dose of ETI-204 (*n* = 14) or placebo (n = 13) in the other (Experiment 23). Survival at 28 days included 8 (61%) ETI-204 and 2 (15%) placebo animals in Experiment 22 and 8 (57%) ETI-204 and 4 placebo (31%) animals in experiment 23. ETI-204 reduced the RR in both experiments (0.45 (0.22, 0.94) and 0.62 (0.31, 1.25), respectively) and this was significant for the former but not the latter experiment (Figure 1). A package insert for ETI-204 refers to the effects of the agent when combined with antibiotics in animal experiments but does not provide specific data [26]. Data regarding the cGMP status of the materials tested was not evident after review of relevant references [26].

### 2.4. Overall Experience with Anti-Toxin Agents

Across the 23 experiments, the three antitoxin agents had effects on the RR on the side of benefit in 16, although only one of these (Experiment 22) was individually significant (Figure 1). In our prior analysis, we found that these effects were consistent in rabbits across Experiments 4 to 11 in the published report with AIGIV, Experiments 16 and 17 in the published report with ETI-204 and Experiments 18 to 21 in the FDA document with ETI-204 (I^2^ = 0% for each) (Figure 2, adapted from Reference [15]). The effects of the agents were also consistent in cynomolgus macaques in the FDA documents across Experiments 13 and 14 with AIGIV and Experiments 22 and 23 with ETI-204 (I^2^ = 0% for each) (Figure 2) [15]. A single RR was calculated for each of these groups of experiments. These combined effects were then analyzed along with RRs from reports with single experiments. The three anti-toxin agents consistently decreased the overall RRs across experiments in rabbits (0.65 (0.50, 0.87), I^2^ = 15.8%), cynomolgus macaques (×0.60 (0.38, 0.93), I^2^ = 25.3%) and when experiments across rabbits and cynomolgus macaques were combined (0.64 (0.51, 0.81), I^2^ = 7.1%) (Figure 3, adapted from Reference [15]).

A review of a smaller group of experiments by investigators at the Centers for Disease Control and Prevention (CDC) had suggested that anti-toxin agents might actually have greater benefit given later after the onset of *B. anthracis* infection and when toxin levels were higher [13]. Although not significant, we found a similar relationship between antitoxin treatment time and survival within and then across those studies with experiments which included both early and late treatment times in the same species [15]. A slope for the relationship between treatment time and the effects of anti-toxin agents on the log RR (slope in log (RR) (95% CI)) was −0.023 (−0.060, 0.015) (*p* = 0.24) with AIG (Experiments 4 to 11) and −0.042 (−0.104, 0.021) (*p* = 0.19) with ETI-204 (Experiments 18 to 21) [15]. When combined, the overall relationship was −0.028 (−0.060, 0.005) (*p* = 0.09) [15]. 

All 23 experiments were based on prospective sample size calculations, 22 were randomized and 20 included prospective observation schedules and euthanasia criteria (Table 3, adapted from Reference [15]). However, only five experiments appeared to be blinded, and as noted previously, none included the hemodynamic support that patients with life-threatening anthrax infection would receive. Survival was the primary endpoint in all studies.

## 3. Effects of the Raxibacumab and AIGIV when Combined with Titrated Hemodynamic Support in a Canine Model of Anthrax Toxin-Associated Shock

We conducted six studies in a canine model, first examining the effects of LT and ET challenges alone (Study 1) or together (Study 2) on survival, hemodynamic function and organ injury, and then the effects of either Raxibacumab (Studies 3, 4 and 5) or AIG (Study 6) when combined with titrated hemodynamic support (HS) on these same parameters (Table 4) [16,17,18,27]. Prior studies suggested that these humanized or human antibodies would bind to canine cells and be effective. One study showed an approximate 81% to 84% sequence identity and similarity respectively, between the sequence alignment of the extracellular domain of human and canine neonatal Fc receptors, and another showed that human IVIG binding to canine lymphocytes and monocytes was inhibited by canine Fc fragments [28,29]. 

Each study we conducted was approved by the National Institutes of Health Clinical Center’s Animal Care and Use Committee [16,17,18,27]. In individual weekly experiments comprising the studies, three or four purpose-bred beagles were continuously sedated, mechanically ventilated via tracheostomies and had systemic, pulmonary and urinary catheters in place. Animals were cared for in a veterinarian intensive care unit by veterinarian technicians and doctors in experiments that were terminated after 96 h of observation. Temperature control and sedation with midazolam, fentanyl and dexmedetomidine were managed uniformly for all groups based on protocols. Ventilator adjustments were made to FiO_2_, positive end-expiratory pressure (PEEP) and ventilator rate based on protocols and continuous pulse oximetry and arterial blood gases (ABGs) were performed at regular intervals. Additional care for all animals included fixed doses of maintenance fluids, gastrointestinal and deep venous thrombosis prophylaxis, and ceftriaxone to prevent catheter-related infections. 

After baseline measurements, animals were challenged with 24 h continuous infusions of LT, ET, LT + ET or, as control for Studies 1 and 2, PA alone. The toxin components used were recombinant molecules prepared as previously described [16]. Toxin doses were reported as the amount of LF or EF used. In studies with Raxibacumab and AIGIV, animals were randomized to treatment or placebo, and personnel caring for the animals were blinded to the treatment assignments. For Raxibacumab, the placebo was an inactive antibody that did not react with LT, ET or PA, and for AIGIV, the placebo was human intravenous immunoglobulin. 

**Figure 4:**
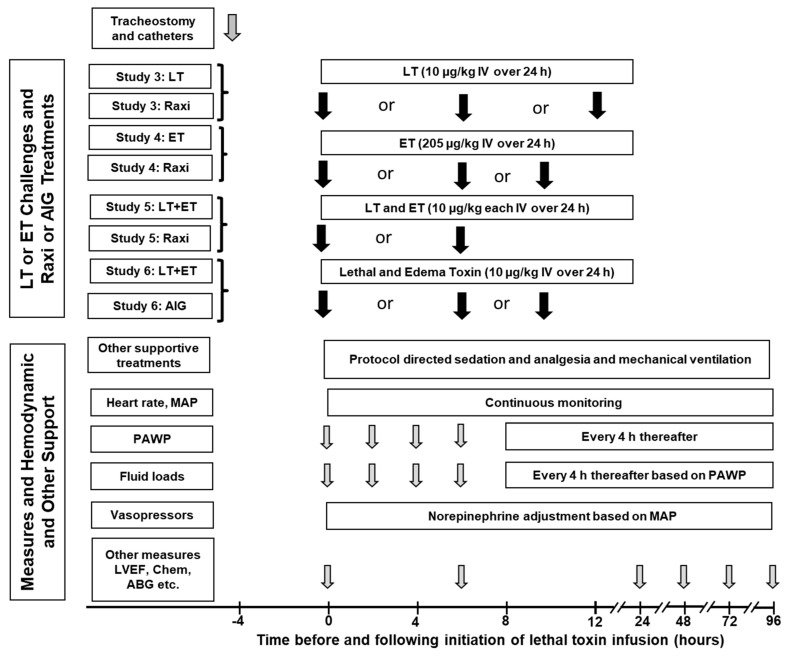
This figure shows for Studies 3 to 6 the timeline of administration of lethal or edema toxins (LT or ET) alone or together, the times of treatment with Raxibacumab (Raxi) or Anthrax Immune Globulin (AIG) and with hemodynamic support (fluids titrated to pulmonary artery wedge pressure and norepinephrine titrated to mean arterial blood pressure), other supportive measures based on established protocols and administered to all animals including sedation and analgesia (fentanyl, midazolam and medetomidine) and mechanical ventilation, and the timing of hemodynamic and other laboratory measures. Raxibacumab was administered as a bolus at the timepoint indicated. The first 50% of the dose of AIG was infused over the 4 h before and the second 50% infused over the 2 h following the designated treatment time. See text for other supportive treatments administered to all animals. Although not shown in the figure, total fluid intake was recorded every 2 h, while urine output was recorded every 24 h and at time of death (LVEF—left ventricular ejection fraction; Chem—chemistries including electrolytes, blood urea nitrogen, creatinine, alanine and aspartate amino-transferases; ABG—arterial blood gases).

In Studies 3 to 6, which included titrated hemodynamic support (HS), to ensure all studied animals had similar preloads at the outset before toxin infusion, pulmonary artery wedge pressures (PAWP) were measured and if <10 mmHg, 1 to 3 boluses (20 mL/kg) of normal saline were administered until a PAWP of at least 10 mmHg was achieved [17,18,27]. For the remainder of studies, animals assigned to groups with HS received a single bolus of 20 mL/kg of normal saline if the PAWP (checked every 2 h for the first 8 h and every 4 h thereafter) was found to be <10 mmHg (Figure 4). Additionally, if at any time mean arterial blood pressure (MAP) decreased to <80 mmHg for >5 min, a norepinephrine infusion was initiated at 0.2 μg/kg/min and, if necessary, increased in a stepwise fashion every 5 min to 0.6, 1 or a maximum of 2 μg/kg/min (and similarly titrated down if MAP was >100 mmHg for >5 min). Amounts of fluid and norepinephrine received by each animal were recorded every 2 h. Control animals in Studies 3 and 4 not receiving HS had hemodynamic measurements performed and recorded, but did not receive titrated fluid boluses or norepinephrine. All supportive therapies were administered by technicians blinded to treatment allocations. 

In an initial dose finding study (Study 1), it was determined that the LD50 doses for LT and ET were 8.4 and 205 µg/kg respectively, administered as continuous infusions over 24 h [16]. Compared to non-toxin controls, lethal doses of either toxin reduced mean arterial blood pressure (MAP), central venous pressure (CVP) and systemic vascular resistance index (SVRI), and increased heart rate (HR), either over the entire 96 h observation period or at early or later time points (*p* ≤ 0.003 overall or for the time interactions). Changes in CVP, HR and SVRI were greater with ET than LT (*p* ≤ 0.02). Only LT progressively reduced left ventricular ejection fraction (LVEF, *p* = 0.0001 for the time interaction). Both toxins progressively increased blood urea nitrogen (BUN), creatinine (Cr) and aspartate and alanine aminotransferases (ALT and AST) and decreased arterial pH and arterial base excess (ABE) (*p* ≤ 0.01). Thus, both toxins when administered in lethal doses produced shock and organ injury over the 96 h observation period. 

Study 2 examined the effects of the two toxins together, administered in equal molar doses of 8.4 µg/kg, the LD50 LT dose [16]. Compared to non-toxin controls (9 of 9 survivors), all ET animals (4 of 4) survived, whereas only 3 of 8 LT animals survived in a pattern different from controls (*p* = 0.01). No animal receiving ET and LT together (0 of 8) survived in patterns that differed both from controls and LT alone (*p* < 0.0001 and 0.05, respectively). The toxins together decreased SVRI (*p* = 0.01), increased HR and reduced CVP and LVEF (*p* ≤ 0.04). The combination also increased Cr, AST, ALT and decreased pH and arterial base excess (ABE) (*p* ≤ 0.05).

**Figure 5:**
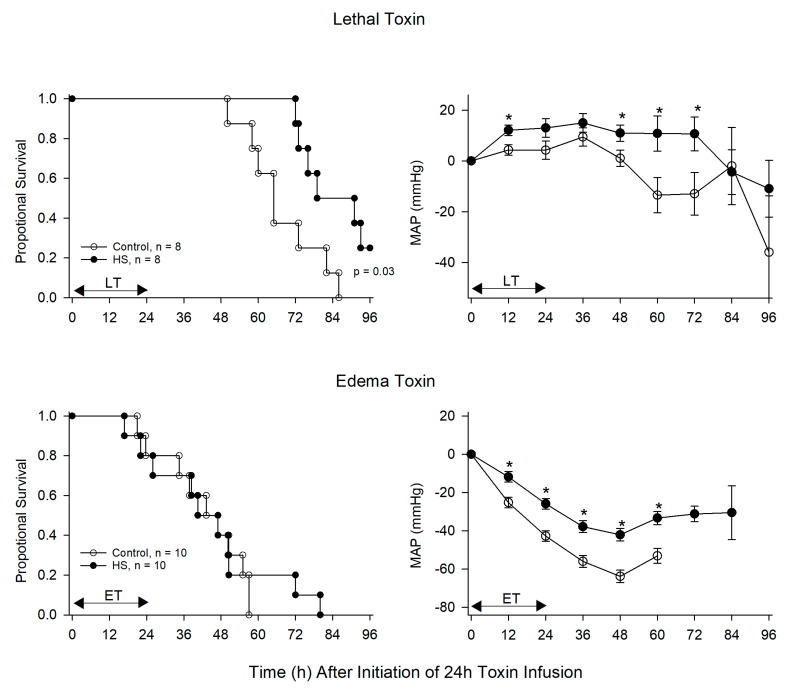
This figure compares the effects of titrated hemodynamic support alone (HS) versus no HS (control) on the proportion of animals surviving and serial mean (±SEM) changes in mean arterial blood pressure (MAP) in animals following the start of 24 h infusions of lethal toxin (LT, upper panels) or edema toxin (ET, lower panels). * *p* ≤ 0.05.

**Figure 6:**
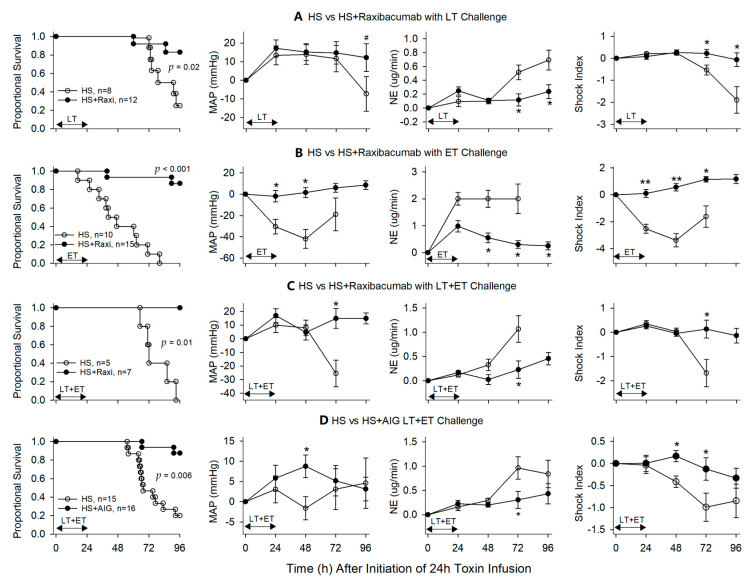
This figure compares the effects of titrated hemodynamic support (HS) alone versus HS with either Raxibacumab or AIG on the proportion of animals surviving and serial mean (±SEM) changes in mean arterial blood pressure (MAP, mmHg), norepinephrine dose (NE, µg/min) and shock index following the start of 24 h infusions of lethal toxin (LT), edema toxin (ET) or LT + ET. This figure combines data for studies in which Raxibacumab was administered at 0, 6 and 12 h with LT (**A**), 0 and 6 h with ET (**B**) and LT + ET (**C**), and AIG was administered at 0, 6 and 9 h with LT + ET (**D**). * *p* ≤ 0.05, ** *p*≤0.001, ^#^ *p* ≤ 0.1.

**Figure 7:**
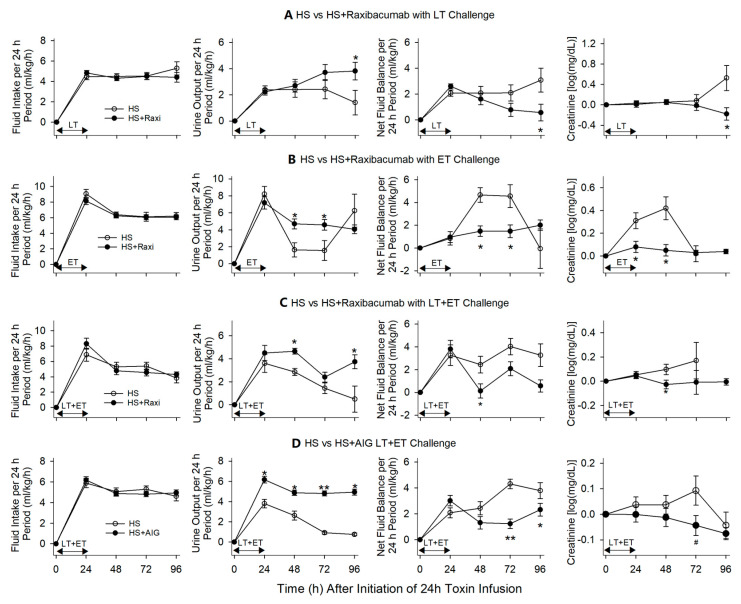
This figure compares the effects of titrated hemodynamic support (HS) alone versus HS with either Raxibacumab or AIG on mean (±SEM) fluid intake, output and net fluid balance over 24 h periods, and mean (±SEM) serial changes in serum creatinine following the start of 24 h infusions of lethal toxin (LT), edema toxin (ET) or LT + ET. This figure combines data for studies in which Raxibacumab was administered at 0, 6 and 12 h with LT (**A**), 0 and 6 h with ET (**B**) and LT + ET (**C**), and AIG was administered at 0, 6 and 9 h with LT + ET (**D**). * *p* ≤ 0.05, ** *p* ≤ 0.001, ^#^ *p* ≤ 0.1.

**Figure 8:**
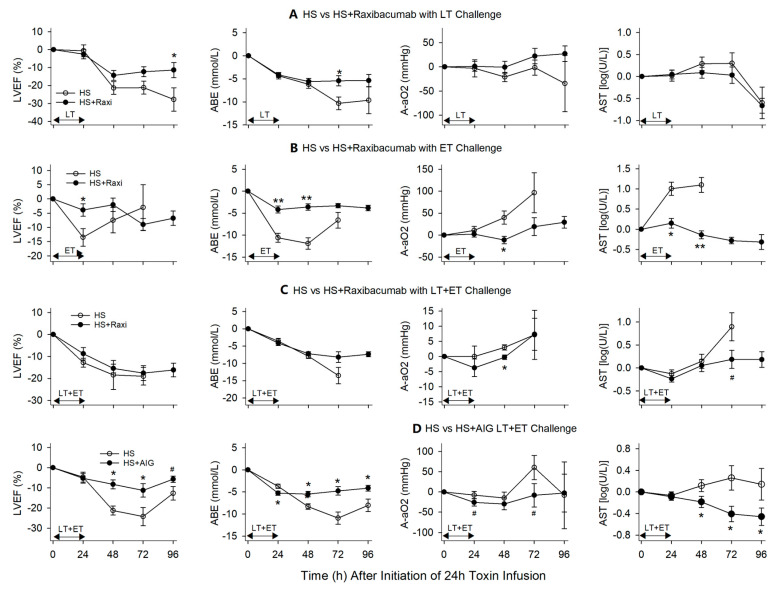
This figure compares the effects of titrated hemodynamic support (HS) alone versus HS with either Raxibacumab or AIG on serial mean (±SEM) changes in left ventricular ejection fraction (LVEF), arterial base excess (ABE), alveolar to arterial oxygen gradient (A-aO_2_) and aspartate aminotransferase (AST) following the start of 24 h infusions of lethal toxin (LT), edema toxin (ET) or LT + ET. This figure combines data for studies in which Raxibacumab was administered at 0, 6 and 12 h with LT (**A**), 0 and 6 h with ET (**B**) and LT + ET (**C**), and AIG was administered at 0, 6 and 9 h with LT + ET (**D**). * *p* ≤ 0.05, ** *p* ≤ 0.001, ^#^ *p* ≤ 0.1.

A timeline (Figure 4) shows for Studies 3 to 6 what toxin and anti-toxin treatments were employed, the timing of anti-toxin treatment and when measures were conducted. Study 3 assessed the effect of Raxibacumab and titrated HS (designated mAb and HS respectively, in Figure 5, Figure 6, Figure 7 and Figure 8) when administered alone or together in animals challenged with lethal 24 h LT challenges across 10 weekly experiments [17]. Hemodynamic support was initiated as needed following the start of toxin challenge and Raxibacumab was administered at time 0, 9 or 12 h as a single 2 mL injection at 10× the molar dose of PA, to each animal [17,27]. Compared to no HS treatment, HS alone increased survival and MAP (*p* ≤ 0.03) (Figure 5). Raxibacumab alone increased survival when administered at 0 or 6 h but not at 12 h (*p* = 0.004 for the time interaction) (data not shown). Compared to HS alone, when Raxibacumab was added to HS at 0, 6 or 12 h, the combination improved survival for each treatment time (*p* ≤ 0.02 when averaged over the 10 experiments) (Figure 6). When other data was combined across the three treatment times, Raxibacumab decreased the amount of norepinephrine animals required and improved the shock index score (SI), which calculates the difference between the normalized values of MAP and norepinephrine usage (a low MAP with increased norepinephrine usage would produce a low SI and a high MAP with decreased norepinephrine usage would produce a high score) (Figure 6). Raxibacumab added to HS also increased urine output, decreased net fluid balance and creatinine and increased arterial base excess (ABE) and left ventricular ejection fraction (LVEF) at one or more time points (*p* ≤ 0.05) (Figure 7 and Figure 8).

Study 4 examined the effects of no treatment and titrated HS alone or together with Raxibacumab in animals challenged with 24 h ET infusions across 10 weekly experiments [27]. Raxibacumab was added to HS at time 0, 6 or 12 h. When examined across the 10 experiments, compared to no treatment, HS alone did not increase survival significantly and most animals had expired by 48 h (*p* = 0.61) (Figure 5). However, HS did increase MAP across multiple time points (Figure 5). Compared to HS alone, Raxibacumab added to HS at any of the three time points increased survival and these increases were significant with treatment at 0 or 6 h (*p* ≤ 0.02). When data was combined across these two earlier treatment times, Raxibacumab added to HS improved survival, reduced norepinephrine requirements and improved the SI (Figure 6). Additionally, Raxibacumab increased urine output, decreased net fluid balance and creatinine, increased LVEF and ABE and decreased the alveolar to arterial oxygen gradient (AaO_2_), ALT and AST at one or more time points (*p* ≤ 0.05) (Figure 7 and Figure 8). 

Study 5 investigated the effects Raxibacumab when added to titrated HS in animals challenged with 24 h infusions of LT and ET together across three weekly experiments including four animals per group [27]. All animals received titrated HS, and Raxibacumab was added to this support at time 0 or 6 h. When examined across the three experiments, no animal receiving HS alone survived, while all animals that had Raxibacumab added to HS survived (*p* = 0.01) (Figure 6). Raxibacumab added to HS also reduced animals’ norepinephrine requirement, improved the SI, increased urine output, reduced net fluid balance, creatinine, AaO_2_ and AST at individual time points (*p* ≤ 0.05 except for AST that was 0.08) (Figure 6, Figure 7 and Figure 8). 

Finally, Study 6 examined the effects of AIG or placebo when added to HS in animals challenged with 24 h infusions of LT and ET together [18]. To reduce hypersensitivity reactions to human protein, AIG and IVIG (the placebo) were administered in gradually escalating doses. Animals received the initial 50% of their treatment dose over 4 h before and the final 50% over 2 h after the designated treatment times of 0, 6 or 9 h. Animals in this study also received diphenhydramine (1 mg/kg, IV) every 6 h for 3 doses and famotidine (1 mg/kg, IV) every 12 h starting with the AIGIV and IVIG treatments. Treatment time points reflect the time that 50% of the total AIG dose was administered. All animals received HS. Whether administered at 0, 6 or 9 h, AIG increased survival and overall had a highly significant survival effect (*p* = 0.006) (Figure 6). Data from the three groups of experiments were also combined for analysis. As with Raxibacumab, AIG reduced norepinephrine requirements, increased the SI and urine output, decreased net fluid balance and creatinine, increased LVEF and ABE and decreased AaO_2_, ALT and AST significantly or in trends approaching significance (*p* ≤ 0.1) (Figure 6, Figure 7 and Figure 8).

## 4. Summary

Septic shock during *B. anthracis* infection is relatively infrequent but appears to be associated with a mortality rate greater than more commonly encountered types of bacterial infection, even when treated in well-resourced medical facilities [1,3]. As part of standard therapy, patients presenting with anthrax-associated sepsis or septic shock will always receive antibiotics and some level of hemodynamic support. Together, the preclinical studies reviewed here present evidence that the anthrax anti-toxin agents now approved by the FDA may add to the beneficial effects of either antibiotics or hemodynamic support during *B. anthracis* infection [15,16,17,18,27]. However, questions remain about the effectiveness of these agents.

Studies examining the effects of the anti-toxin agents when combined with antibiotic therapy during live *B. anthracis* infection had at least two strengths. First, the agents had highly consistent effects on the side of benefit across the species and agents studied. As previously presented, this consistency allowed these results to be combined and to demonstrate a significant overall benefit with treatment [15]. Second, in some experiments, combined anti-toxin and antibiotic treatment had effects on the side of benefit even when started well after bacteria challenge and when infection had become severe enough to result in death [15]. Analysis suggested that anti-toxin treatment might actually have had greater benefit when administered later at a time when toxin levels would have been higher [15]. However, an important weakness in these studies is that anti-toxin treatment was only significantly beneficial in a single experiment, and this was an unblinded one [15]. In fact, only five experiments were reported to have been blinded ones. The resources required to conduct delayed treatment studies with a highly lethal bacteria are clearly substantial. Experiments demonstrating that delayed treatment with either Raxibacumab or AIGIV produced beneficial trends in survival in rabbits required 180 and 336 animals to be initially infected, respectively. We previously conducted a power analysis based on mortality rates at the time just prior to treatment and the overall effect sizes of the anti-toxin agents in these two experiments [15]. For Raxibacumab and AIGIV, it would have required 496 and 1138 animals to be infected respectively, to have sufficient numbers of surviving animals remaining for randomization at the times studied to have an 80% power to detect a 17% or 19% difference in mortality respectively, at a two-sided level of significance of *p* = 0.05 [15]. On the one hand, such studies, especially when requiring Biosafety Level-3 conditions, would be prohibitive. On the other hand, these are the kinds of designs and results that are typically required from clinical trials to approve adjunctive therapies for sepsis [15]. At this time, it is actually not known whether any of these three agents would have significant beneficial effects when combined with antibiotic support in an adequately powered and blinded preclinical trial, much less a clinical one. Little physiologic data, such as measures of hemodynamic or organ function, was available in these studies to support the agents’ beneficial survival trends.

The studies examining the effects of Raxibacumab and AIGIV when combined with titrated hemodynamic support in the toxin-challenged canine model also have strengths [16,17,18]. Most importantly, they were blinded studies conducted in canine subjects that were monitored and treated just as patients presenting with severe anthrax-associated septic shock would be managed. While hemodynamic support alone appeared to have limited survival effects in the model, both anti-toxin agents had strong beneficial effects when added to that support. In contrast to the live *B. anthracis*-challenged models, because these canine studies were done at Biosafety Level-2, considerable hemodynamic and organ function data was available to corroborate the apparent beneficial survival effects of the two agents. One of the most striking findings was that the anti-toxin agents decreased the amount of vasopressor support subjects required while promoting net negative fluid balances. Both of these actions are associated with improved outcomes in patients. Although we previously used an LT-challenged rat model that examined the effects of fluid administration with Raxibacumab in one study and norepinephrine infusion alone in another, these standard treatments were not titrated or administered together. The canine studies we reviewed here are the first we are aware of to examine the effects of titrated hemodynamic support in a model of anthrax toxin-associated shock [30,31]. The most obvious weakness in these canine studies though is that they were toxin- and not bacteria-challenged studies. Shock with *B. anthracis* is probably not just related to toxin production [32,33,34]. For example, the bacterium has a highly bioactive cell wall that produces a robust, maladaptive host inflammatory response [35,36]. This response may make a major contribution to shock and organ injury with *B. anthracis* as it is thought to do with other types of bacteria and would likely not be altered by anti-toxin treatment. Another weakness in the model is that toxin challenge, even though administered over 24 h, is probably more rapid and does not simulate the time course of toxin release during live bacterial infection. Based on how these studies were conducted, it is unclear whether anti-toxin treatment delayed later than 6 to 12 h would still have beneficial effects. 

Ultimately, though, neither of these two groups of studies provides evidence as to whether any of the three now FDA-approved anthrax anti-toxin agents add significant benefit to the standard clinical support for sepsis and septic shock, which includes antibiotic and hemodynamic support administered together [15]. Animal studies with other types of bacteria have suggested that these two therapies have synergistic actions, with hemodynamic therapy supporting tissue perfusion and organ function, while antibiotics begin to promote microbial clearance [12]. Clinical sepsis guidelines direct that both arms of therapy be initiated as quickly as possible together [11]. As noted above, clinical experience with AIGIV was not controlled [1]. In the largest experience with it in the UK anthrax outbreak in injection drug users, it was generally administered to more critically ill patients and there were insufficient untreated patients with comparably severe disease to make reliable comparisons. It is unlikely that these anti-toxin agents will ever be studied in controlled clinical studies. However, large animal bacteria sepsis models do exist that would allow the agents to be studied with standard sepsis therapies. The canine model used here was developed to examine the addition of adjunctive therapies to standard antibiotic and hemodynamic support [37,38,39]. Based on this canine model, a nonhuman primate model is under development that is intended for use at Biosafety Levels-3 and 4 (personal communication with Dr. Daniel Chertow). While resource-intensive, such models could be used to confirm that anthrax anti-toxin agents combined with both antibiotic and hemodynamic support have effects consistent with those when the agents are used with these standard therapies individually. The cost of such studies would appear to outweigh the risk of discovering that these anti-toxin agents are ineffective in patients with severe disease during a large outbreak of *B. anthracis* infection. 

## Figures and Tables

**Table 1:** Study characteristics.

Author (Year)	Exp ^#^	Species	Sex	Inhalational Ames Strain Challenge Dose	Antibiotics Treatment				Anti-Toxin Treatment				Trigger
					Type	Route	Dose	Time (h)	Type	Route	Dose	Time (h)	
Migone (2009) [19]	1	Rabbit	M/F	200 × LD50	Levo	Oral	50 mg/kg	0	RAXI	IV	40 mg/kg	NR	PA det
	2	Cynos	M/F	200 × LD50	Cipro	Oral	75 mg	0	RAXI	IV	40 mg/kg	NR	PA det
Migone (2015) [20]	3	Rabbit	M/F	2.1 × 10^7^ s	Levo	GI, IV	50 mg/kg	84	RAXI	IV	40 mg/kg	84	NT
Kammanadiminti (2014) [22]	4	Rabbit	NR	2.1 × 10^7^ s	Levo	Oral	50 mg/kg	30	AIGIV	IV	15 U/kg	30	NT
	5	Rabbit	NR	2.1 × 10^7^ s	Levo	Oral	50 mg/kg	36	AIGIV	IV	15 U/kg	36	NT
	6	Rabbit	NR	2.1 × 10^7^ s	Levo	Oral	50 mg/kg	48	AIGIV	IV	15 U/kg	48	NT
	7	Rabbit	NR	2.1 × 10^7^ s	Levo	Oral	50 mg/kg	60	AIGIV	IV	15 U/kg	60	NT
	8	Rabbit	NR	2.1 × 10^7^ s	Levo	Oral	50 mg/kg	60	AIGIV	IV	15 U/kg	60	NT
	9	Rabbit	NR	2.1 × 10^7^ s	Levo	Oral	50 mg/kg	72	AIGIV	IV	15 U/kg	72	NT
	10	Rabbit	NR	2.1 × 10^7^ s	Levo	Oral	50 mg/kg	84	AIGIV	IV	15 U/kg	84	NT
	11	Rabbit	NR	2.1 × 10^7^ s	Levo	Oral	50 mg/kg	96	AIGIV	IV	15 U/kg	96	NT
	12	Rabbit	NR	2.1 × 10^7^ s	Levo	Oral	50 mg/kg	96	AIGIV	IV	15 U/kg	96	NT
FDA AIG (2015) [23]	13	Cynos	M/F	200 × LD50	Cipro	Oral	32 mg/kg 16 mg/kg	64	AIGIV	IV	15 U/kg	64	NT
	14	Cynos	M/F	200 × LD50	Cipro	Oral	32 mg/kg 16 mg/kg	64	AIGIV	IV	30 U/kg	64	NT
Biron (2015) [24]	15	Rabbit	M/F	150–250 × LD50	Doxy	IV	2.0 mg/kg	30	ETI	IV	8 mg/kg	30 *	PA det
Yamomato (2016) [25]	16	Rabbit	M/F	200 × LD50	Levo	Oral	50 mg/kg	9	ETI	IV	4 mg/kg	9	NT
	17	Rabbit	M/F	200 × LD50	Levo	Oral	50 mg/kg	9	ETI	IM	8 mg/kg	9	NT
FDA ETI (2015) [26]	18	Rabbit	NR	NR	Levo	NR	50 mg/kg	96	ETI	IV	8 mg/kg	96	NT
	19	Rabbit	NR	NR	Levo	NR	50 mg/kg	72	ETI	IV	8 mg/kg	72	NT
	20	Rabbit	NR	NR	Levo	NR	50 mg/kg	30	ETI	IV	16 mg/kg	30	NT
	21	Rabbit	NR	NR	Levo	Oral	6.5 mg/kg	72	ETI	IV	16 mg/kg	72	NT
	22	Cynos	NR	NR	Cipro	Oral	10 mg/kg	48	ETI	IV	8 mg/kg	48 **	PA det
	23	Cynos	NR	NR	Cipro	Oral	10 mg/kg	48	ETI	IV	8 mg/kg	48 **	PA det

* If no protective antigen (PA) detected, then treatment given at 30 h post-exposure; ** PA detection equivalent to 48 h post-exposure. AIGIV—Anthrax Immune Globulin Intravenous; Cipro—Ciprofloxacin; Cynos—cynomolgus macaques; Doxy—Doxycycline; ETI—ETI-204; Exp ^#^—experiment number; GI—gastrointestinal; IM—intramuscular; IV—intravenous; LD—lethal dose; Levo—Levofloxacin; NR—not recorded; NT—no trigger; PA det—protective antigen detection; RAXI—Raxibacumab; (Table adapted from Reference [15]).

**Table 2:** Summary of treatments, data sources and numbers of animals challenged and randomized or assigned in experiments.

Author (Year)	Experiment Number	Treatments		Data Source	Animal Number		
					Challenged ^##^	Randomized or Assigned	
		Control	Anti-Toxin			Control	Anti-Toxin
Migone (2009) [19]	1	Levo + placebo *	Levo + Raxi	FDA-BD	40	20	20
	2	Cipro + placebo *	Cipro + Raxi	FDA-BD	28	14	14
Migone (2015) [20]	3	Levo + Raxi-buffer	Levo + Raxi	PR	180	37	39
Kammanadiminti (2014) [22]	4	Levo + IGIV	Levo + AIG	PR	16	8	8
	5	Levo + IGIV	Levo + AIG	PR	16	8	7
	6	Levo + IGIV	Levo + AIG	PR	16	8	8
	7	Levo + IGIV	Levo + AIG	PR	16	8	8
	8	Levo + IGIV	Levo + AIG	PR	20	10	8
	9	Levo + IGIV	Levo + AIG	PR	72	20	23
	10	Levo + IGIV	Levo + AIG	PR	19	9	10
	11	Levo + IGIV	Levo + AIG	PR	72	8	7
FDA AIG (2015) [23]	12	Levo + IGIV	Levo + AIG	FDA-BD	336	33	31
	13	Cipro + IGIV	Cipro + AIG	FDA-BD	20	12 ^#^	12 ^#^
	14	Cipro + IGIV	Cipro + AIG	FDA-BD	20	12 ^#^	14 ^#^
Biron (2015) [24]	15	Doxy + Saline	Doxy + ETI	PR	20	10	10
Yamomato (2016) [25]	16	Levo **	Levo + ETI	PR	21	12	9
	17	Levo **	Levo + ETI	PR	21	12	9
FDA ETI (2015) [26]	18	Levo **	Levo + ETI	FDA-BD	32	5	4
	19	Levo **	Levo + ETI	FDA-BD	32	9	11
	20	Levo **	Levo + ETI	FDA-BD	40	20	20
	21	Levo **	Levo + ETI	FDA-BD	103	38	34
	22	Cipro **	Cipro + ETI	FDA-BD	32	13	13
	23	Cipro **	Cipro + ETI	FDA-BD	32	13	14

* Placebo noted but not described; ** No Placebo described; ^#^ Animals that had not died but were bacteremic at 64 h; ^##^ Number of animals infected at the outset of the experiment, from which some expired prior to later treatment in several experiments; Cipro—ciprofloxacin; FDA-BD—Food and Drug Administration briefing document; IGIV—Human Immune Globulin Intravenous; Levo—levofloxacin; PR—published results; Raxi—Raxibacumab (Table adapted from Reference [15]).

**Table 3:** Study designs.

Author (Year)	Exp ^#^	Agent	Species	Random.	Blind.	Pro. Samp. Size	Pro. Obs. Sched.	Pro. Euth. Crit.
Migone (2009) [19]	1	RAXI	Rabbit	Yes	Yes	Yes	NR	NR
	2	RAXI	Cynos	Yes	Yes	Yes	NR	NR
Migone (2015) [20]	3	RAXI	Rabbit	Yes	Yes	Yes	NR	NR
Kammanadiminti (2014) [22]	4	AIG	Rabbit	Yes	No	Yes	Yes	Yes
	5	AIG	Rabbit	Yes	No	Yes	Yes	Yes
	6	AIG	Rabbit	Yes	No	Yes	Yes	Yes
	7	AIG	Rabbit	Yes	No	Yes	Yes	Yes
	8	AIG	Rabbit	Yes	No	Yes	Yes	Yes
	9	AIG	Rabbit	Yes	No	Yes	Yes	Yes
	10	AIG	Rabbit	Yes	No	Yes	Yes	Yes
	11	AIG	Rabbit	Yes	No	Yes	Yes	Yes
FDA AIG (2015) [23]	12	AIG	Rabbit	Yes	Yes	Yes	Yes	Yes
	13	AIG	Cynos	Yes	No	Yes	Yes	Yes
	14	AIG	Cynos	Yes	No	Yes	Yes	Yes
Biron (2015) [24]	15	ETI	Rabbit	No	No	Yes	Yes	Yes
Yamomato (2016) [25]	16	ETI	Rabbit	Yes	No	Yes	Yes	Yes
	17	ETI	Rabbit	Yes	No	Yes	Yes	Yes
FDA ETI (2015) [26]	18	ETI	Rabbit	Yes	No	Yes	Yes	Yes
	19	ETI	Rabbit	Yes	No	Yes	Yes	Yes
	20	ETI	Rabbit	Yes	No	Yes	Yes	Yes
	21	ETI	Rabbit	Yes	No	Yes	Yes	Yes
	22	ETI	Cynos	Yes	No	Yes	Yes	Yes
	23	ETI	Cynos	Yes	No	Yes	Yes	Yes

Blind.—blinding; Euth. crit.—prospective euthanasia criteria; Cynos—cynomolgus macaques; Exp. #—experiment number; NR—not reported; Pro. obs. sched.—prospective observation schedule; Pro. samp. size—Prospective sample size analysis; Random.—randomization; (Table adapted from Reference [15]).

**Table 4:** Summary of studies.

Report	Study	Challenge	Treatment Comparisons	Timing of Anti-Toxin Treatments *	Purpose of Study
Sweeney (2010) [16]	1	LT, ET or Diluent	None	NA	Investigate the survival and cardiopulmonary effects of LT or ET alone versus a diluent challenge
	2	LT, ET, LT + ET or Diluent	None	NA	Investigate the survival and cardiopulmonary effects of LT + ET together versus a diluent challenge
Barochia (2012) [17]	3	LT	HS ** vs. no HS	NA	Investigate the survival effects of HS alone versus no HS support with LT
			Raxi + HS vs. HS alone	0, 9, or 12 h	Investigate the survival effects of Raxi versus HS with LT
Remy (2015) [27]	4	ET	HS vs. no HS	NA	Investigate the survival effects of HS alone versus no HS with ET
			Raxi + HS vs. HS alone	0, 6, or 12 h	Investigate the survival effects of Raxi with HS versus HS alone with ET
	5	LT + ET	Raxi + HS vs. HS alone	0 or 6 h	Investigate the survival effects of Raxi with HS versus HS alone with LT + ET
Suffredini (2017) [18]	6	LT + ET	AIG-IV + HS vs. HS alone	0, 6 or 9 h	Investigate the survival effects of AIG-IV with HS versus HS alone with LT + ET

AIG-IV—Anthrax Immune Globulin-Intravenous; ET—edema toxin; HS—hemodynamic support; LT—lethal toxin; NA—not applicable; Raxi—Raxibacumab. * Antitoxin treatment was administered at the start of the 24 h toxin infusion (0 h) or 6, 9 or 12 h after toxin infusion was started; ** Hemodynamic support was initiated with the start of the 24 h toxin infusion and then continued as needed for the 96 h of study and consisted of normal saline administered based on pulmonary arterial wedge pressures and norepinephrine titrated based on mean arterial blood pressure.
